# Eco-friendly solutions: a comprehensive review of natural coagulants for sustainable water treatment

**DOI:** 10.1007/s10653-025-02803-3

**Published:** 2025-10-31

**Authors:** Sayed Kotb Ali, Moaz M. Abdou, Mostafa M. Emara, Rabie Saad Farag, Mahmoud F. Mubarak

**Affiliations:** 1 Department of Chemistry, Faculty of Science, Al-Azhar University, Nasr City, Cairo, 11884 Egypt; 2 Egyptian Petroleum Research Institute, Nasr City, Cairo, 11727 Egypt

**Keywords:** Coagulation, Natural coagulants, Chemical coagulants, Water treatment, Turbidity removal, Environmental sustainability

## Abstract

Water pollution and scarcity are escalating global challenges that necessitate the development of sustainable water treatment technologies. Coagulation-flocculation, a crucial method for water purification, has traditionally relied on chemical coagulants such as alum and ferric chloride. However, these chemical treatments are associated with significant drawbacks, including the generation of toxic sludge, disruption of water pH, high operational costs, and adverse environmental impacts. In response to these issues, natural coagulants derived from plants, animals, and microorganisms have emerged as promising eco-friendly alternatives. This review explores the mechanisms, applications, and environmental advantages of natural coagulants in water treatment. Studies demonstrate their effectiveness in removing turbidity, heavy metals, pathogens, and other pollutants from both surface and synthetic wastewater. Compared to chemical coagulants, natural alternatives produce less sludge, are less toxic, and offer a more sustainable and environmentally friendly solution to water treatment. However, challenges persist, particularly in terms of scalability, standardization, and cost-effectiveness. The performance of natural coagulants can vary depending on geographical origin, and the lack of standardized extraction methods hinders their large-scale application. Future research should focus on optimizing extraction processes, addressing scalability issues, and exploring hybrid systems that combine the benefits of both natural and chemical coagulants. The continued development of natural coagulants holds great promise for creating more sustainable, cost-effective, and environmentally friendly water treatment solutions.

**Graphical Abstract:**

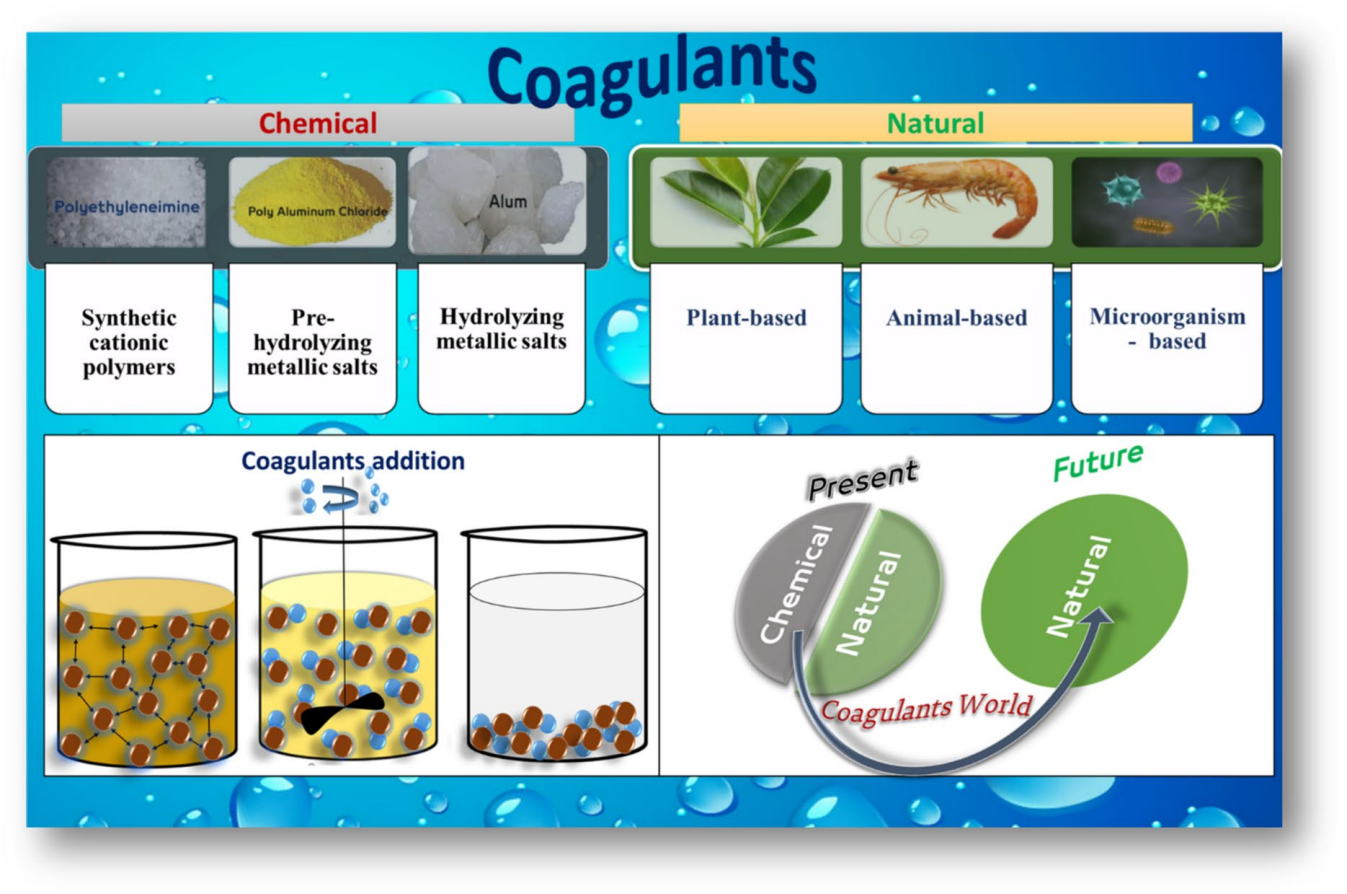

## Introduction

Water is fundamental to supporting life and plays a vital role as a primary resource in energy generation (Ehtisham et al., 2024), agriculture, industrial production, and various other economic activities. It plays a crucial role in transforming oceans, seas, rivers, and lakes through the hydrological cycle, ensuring the preservation of ecological balance, fostering economic growth, and supporting human activities. There is no doubt that the availability of dependable water resources is fundamentally connected to both human survival and the preservation of ecosystems. Access to safe and sustainable drinking water is increasingly becoming a pressing challenge for nations across the globe (Ho et al., 2025). Rapid urbanization, industrialization, and population growth have polluted surface water bodies. There are several reasons for the alarmingly high concentrations of toxic chemicals, inorganic nutrients, and pathogens in surface water flowing through urban landscapes (Badawi et al., 2024a; Kanwal et al., 2025; Wang et al., 2024). They range from land erosion, stormwater runoff, and industrial wastewater, to agricultural effluent (Kakoi et al., 2016). This is contributing to water scarcity in numerous regions across the globe, following known forecasts (Emenike et al., 2022). By this year: (2025), the United Nations forecasts that nearly half of the world's population will live in areas experiencing acute water shortages (Saeed-Ul-Hassan et al., 2024). Water scarcity poses a dual challenge, endangering both human health and food security, economic progress, and environmental sustainability (Ahmed et al., 2023; Badawi et al., 2024b; Salama et al., 2024). Furthermore, pollution from industrial and agricultural activities, as well as wastewater discharge from domestic sources, significantly degrades the quality of water (Hassan et al., 2024). These pollutants harm water resources, making it essential to tackle water pollution as a critical environmental concern (Al Alwan et al., 2024; Hassaan et al., 2023). Creating sustainable and economically feasible water treatment technologies is vital to effectively tackling these difficulties. Coagulation-flocculation is a method used in water treatment, where colloidal particles can be destabilized by adding coagulants/flocculants. This method creates larger aggregates, which can be then efficiently removed through sedimentation or filtration techniques. (Benalia et al., 2024a). Coagulation and flocculation are preferred water treatment methods because they are cost-efficient, simple to operate, and highly effective in eliminating contaminants from water. The performance and success of the coagulation process are heavily influenced by the selection and type of coagulant used. (Ibrahim et al., 2021). Chemical coagulants can be categorized into: organic and inorganic coagulants. Inorganic coagulants are typically aluminium or iron-based salts; common inorganic coagulants are iron and aluminium salts, because of their excellent effectiveness in removing pollutants, simplicity of application, and affordability. (Badawi et al., 2024c; Ibrahim et al., 2021). The application of these coagulants presents several challenges and drawbacks, such as generating large amounts of sludge (Badawi & Hassan, 2024; Choksi et al., 2023), the need to regulate alkalinity and pH levels, and the presence of high residual metal concentrations in the treated water (Benalia & Derbal, 2015). The presence of aluminium ions in water has been associated with human health concerns, including Alzheimer’s disease (Lacang & Ligaray, 2023; Wang et al., 2016). As conventional chemical coagulants contribute to the environmental burden, there is a growing interest in exploring sustainable and eco-friendly alternatives, such as natural coagulants (Badawi et al., 2025). Using natural coagulants works similarly to chemical coagulants by clumping colloidal particles together, but it generates less biodegradable sludge that can be repurposed as fertilizer. Additionally, it eliminates the need for pH and alkalinity adjustments (Tomasi et al., 2025).

Despite the promising potential of natural coagulants, comprehensive reviews that assess their feasibility and the challenges of implementing them in large-scale water treatment are still lacking. This review aims to fill that gap by providing an overview of the benefits and key challenges associated with the use of bio-coagulants. It seeks to encourage further research and development in this field, contributing to the growing body of knowledge on eco-friendly and sustainable water treatment technologies. Ultimately, the goal is to help meet the increasing global demand for safe drinking water while minimizing environmental impact.

## Fundamentals of colloids in water systems

### The colloidal state

**Fig. 1:**
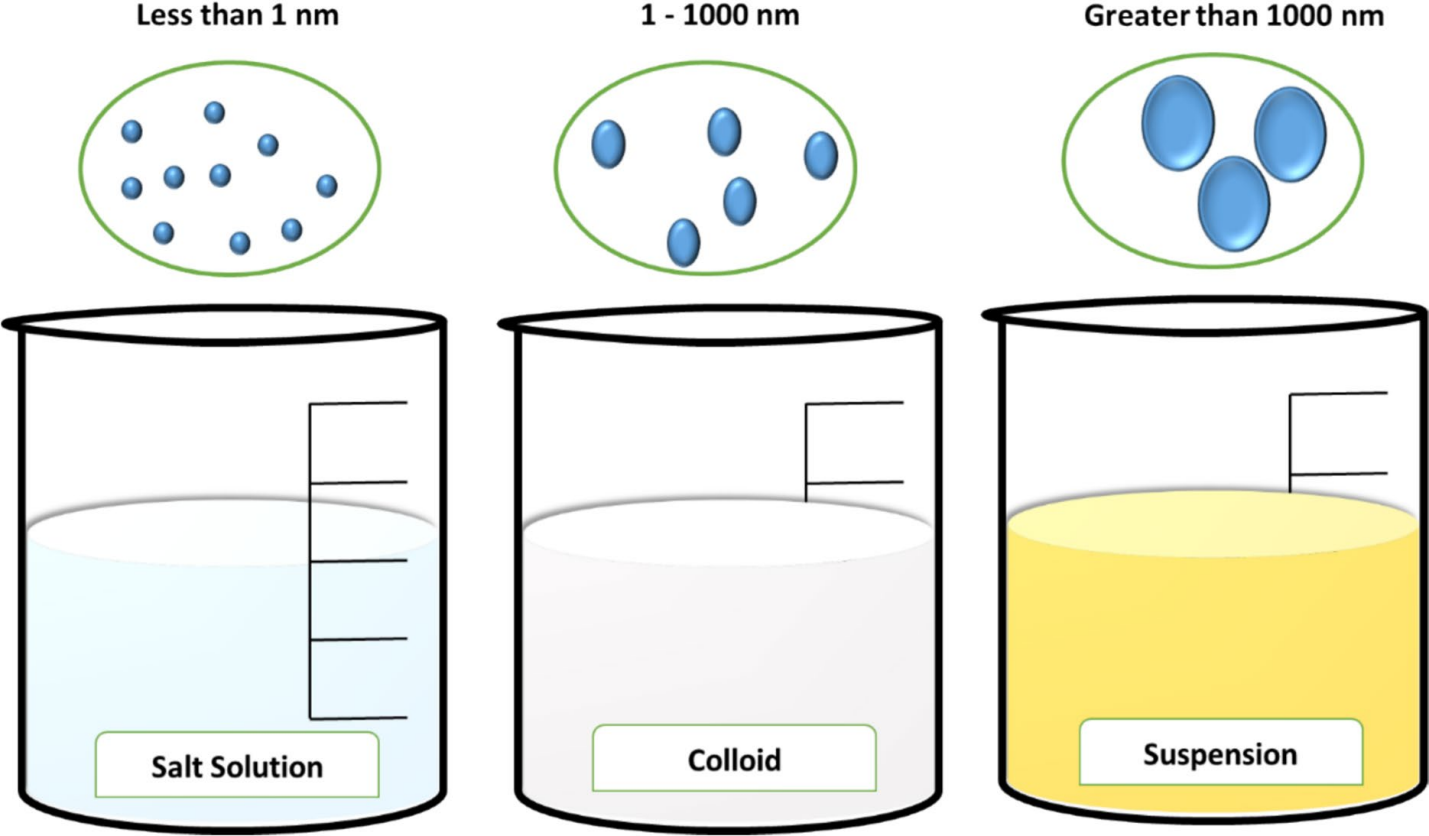
Representation of true solution, colloid, and suspension with their characteristic particle size ranges *Source*: Author’s own illustration

Substances in water and wastewater originate from land erosion, mineral dissolution, decaying vegetation, and discharges from domestic and industrial sources. In any water or wastewater, these substances may include suspended or dissolved organic and inorganic materials, as well as various biological entities like bacteria, algae, and viruses. Water and wastewater contain a great deal of suspended material that is microscopic or submicroscopic in size. Particles size that fall within the range (1–1000 nm) are referred to as colloids (Fig. 1 and Table 1). Colloidal particles contribute to higher turbidity, color, and chemical oxygen demand (COD) in water. The minute size of colloidal particles (< 1 μm) (< 1000 nm) results in significantly slower sedimentation rates compared to their rate of diffusion, as long as repulsive forces between particles prevent aggregation, particles remain stable (Suopajärvi, 2015). For colloids, surface properties outweigh gravitational forces. These surface characteristics prevent colloids from aggregating and becoming sufficiently heavy to settle under gravity. For example, “a 1-micron colloid would take 1 year to settle (by gravity) a distance of 1 foot”. In addition, colloids are generally too small to be caught by standard filtration equipment. Because the force of gravity on them is minimal, they will neither settle nor be filtered until they combine in clumps of larger particles (El-taweel et al., 2023; Kamphorst et al., 2024).

**Table 1:** Difference between true, colloidal and suspended solutions

Solution type	True solution	Colloidal solution	Suspension
Particle size	< 1 nm	1–1000 nm	> 1000 nm
Visibility	Invisible	Visible under ultramicroscope	Visible to the naked eye
Settling	No	No	Yes
Tyndall effect	Absent	Present	Absent

The term “colloid” has already been in use since 1862 to describe liquids that consist of colloidal dispersions. The idea of the colloidal state was originally observed by Thomas Graham in 1861 (Vilela et al., 2018). Half a century later, Wolfgang Ostwald described the colloidal state as a "world of neglected dimensions," highlighting the domain of systems in which particles are exceedingly small (Florence & Attwood, 2015).

The particulate matter in a colloidal solution scatters the light and prevents its direct transmission through it. This phenomenon, which was investigated and documented by John Tyndall, is called the “Tyndall Effect.” The “Tyndall effect” gives a simple way of determining if a given mixture is colloidal or not.

**Fig. 2:**
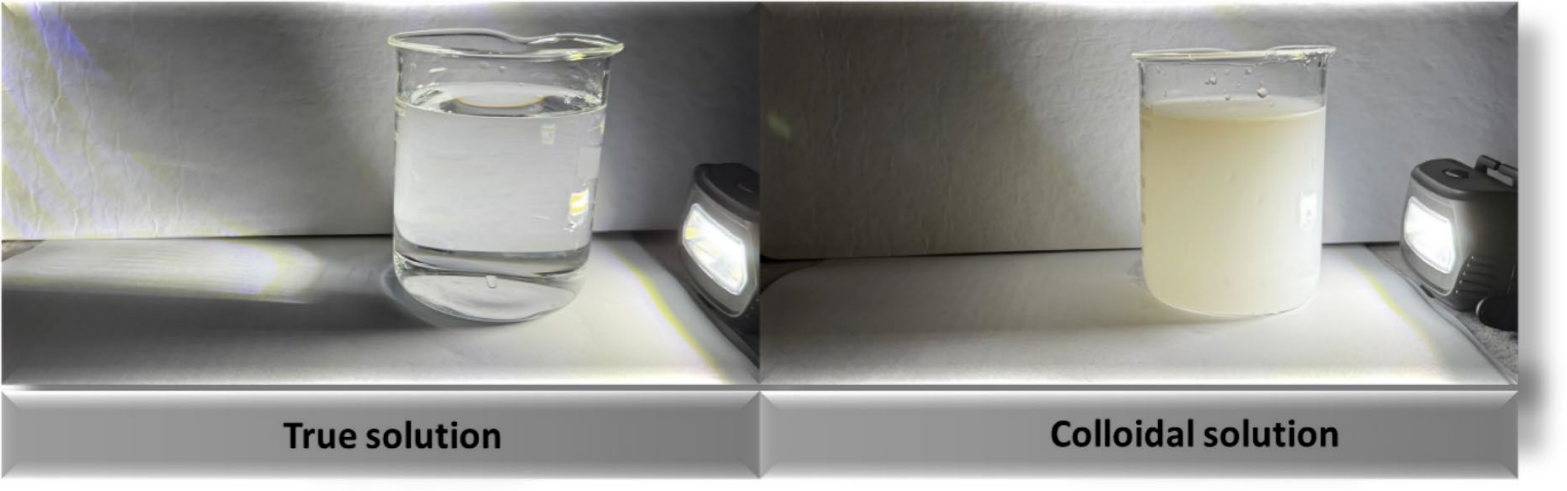
Demonstration of the Tyndall effect: light passes through a true solution (left) without scattering, while scattering is observed in a colloidal solution (right). *Source*: Author’s own photograph

The “Tyndall Effect” refers to the phenomenon where light passes cleanly through a true solution but is scattered in all directions by the dispersed particles in a colloidal solution, making the light visible. This scattering distinguishes colloidal mixtures from true solutions (Vilela et al., 2018), (Fig. 2). The “Tyndall effect” has been explored in various research studies focusing on water quality monitoring, notable examples include: arsenic removal monitored by the “Tyndall effect” (Vera-Aguilar et al., 2015), and the relationship between tea concentration and the “Tyndall effect” (Yang, et al., 2023). These studies highlight the diverse applications of the “Tyndall effect” in monitoring and assessing water quality, demonstrating its utility in detecting contaminants and analyzing colloidal suspensions.

The “Tyndall Effect” is one of the elementary properties of colloidal systems by which they can be differentiated from true solutions. Due to their small particle size, their continuous random motion is caused by impacts on the molecules of the medium of dispersion. It was the botanist Robert Brown who initially noticed the random, zigzag movement called Brownian motion in 1827. The phenomenon of Brownian movement entails that extremely small particles in suspension in a liquid move in what appear to be random pathways, although the liquid itself exhibits laminar flow characteristics. The Brownian motion phenomenon in colloidal solutions arises due to interactions between the dispersion medium molecules and the colloidal particles. It is assumed that the interactions between the colloidal particles and the molecules of the dispersive medium are random (Petsev & Emulsions, 2004), resulting in the zig-zag (random) motion of the colloidal particles, a phenomenon that was mathematically analyzed by Smoluchowski (Smoluchowski, 1917). Another characteristic of colloidal systems is that they maintain particles in suspension through this motion. Absorption is another key feature of colloids since the finely divided colloidal particles have a large surface area. However, colloidal particles have minimal impact on the colligative properties of the colloidal solution, such as boiling point and freezing point (Lagasse, 2000).

### Colloidal stability and brownian motion

Colloids are typically described as water-suspended particles with the ability to move but not as solutes dissolved completely. Commonly encountered colloids in environmental systems are clay minerals, metal oxides, bacteria, viruses, and organic macromolecules. Since they are chemically heterogeneous, colloids have special properties and behavior; thus, they cannot be simply separated from the traditional division of “dissolved” and “solid” phases. Due to their high specific surface area (SSA) and high density of reactive surface functional groups, colloids serve as vital carriers for important substances such as organic compounds, macronutrients, micronutrients, heavy metal ions, and organic pollutants.

Consequently, they play a critical role in determining groundwater and surface water quality (Spielman-Sun et al., 2024). The colloid's stability depends on their surface properties, they are categorized into two broad categories: lyophobic and lyophilic, or in the context of water treatment, hydrophobic and hydrophilic. Colloids exhibit a strikingly high ratio of surface area to mass (Bratby, 2016), in contrast to hydrophobic colloids, hydrophilic colloids are more difficult to aggregate due to their interaction with water. The charge strength of colloids depends on their quality, pH, temperature, and the ionic strength of the solvent. In an aqueous environment, colloidal particles typically gain a negative primary charge, generating repulsive forces that keep them separated and prevent them from aggregating (Bratby, 2016).

**Fig. 3:**
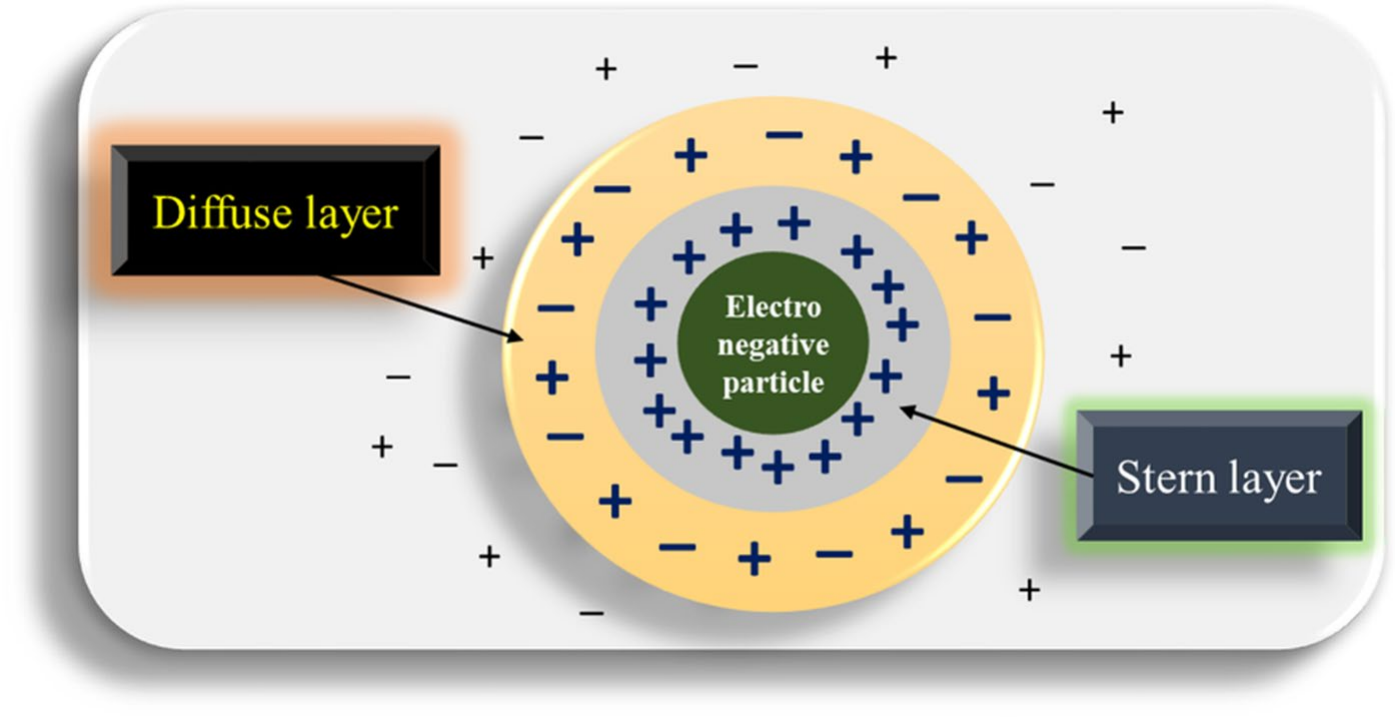
Diagrammatic illustration of the electric double-layer

During the suspension of colloids in liquids, the surface charge of the colloids affects the ion distribution within the liquid, resulting in a higher concentration of counter-ions near the surfaces compared to the bulk. This concentration decreases as the distance from the particle surface increases. Electrical Double Layer (EDL) is formed by thermal motion (Brownian motion) and ionic repulsion or attraction, consisting of a charged surface and a neutralizing excess of counter-ions over co-ions, distributed diffusely in the surrounding liquid (Fig. 3). An electrical double layer (EDL) forms when a charged surface comes in contact with liquid. Coagulation and flocculation are connected with high molecular weight macromolecules, *e.g.,* polymers and humic substances, or metal oxy-hydroxide precipitates (Trefalt et al., 2016).

The EDL can be visualized as two parallel layers of charge extending from the surface. The first, the 'stern layer,' made up of the charge accumulated at the surface, can either be positive or negative, based on the characteristics of the surface (Hernández-Martínez et al., 2018). Ion adsorption or speciation changes of the functional groups may result in a charge change of the stern layer. The second layer consists of ‘free’ ions that are attracted to the surface charge through the Coulomb force, thus adding to the electrical neutralization of the stern layer (Jalil & Pyell, 2018). In comparison with the stern layer, where ions are tightly bound, ions in this second layer possess greater mobility in the fluid around them since they are also subject to the influences of thermal motion and electrostatic forces. The second layer is sometimes called the "diffuse layer” (Fig. 3), a very important layer in colloidal systems due to its very high surface-area-to-volume ratio (Jalil & Pyell, 2018). Factors that influence the EDL can, therefore, significantly impact the behavior of these systems.

### Van der Waals forces

The van der Waals force is a distance-dependent, weak interaction between atoms or molecules and does not cause chemical bonding. The van der Waals force increases with an increase in the size of a molecule, and therefore it is a factor that needs to be kept in mind while dealing with colloidal systems (Kawai et al., 2016). When colloidal particles move close to each other, they are within a distance referred to as the van der Waals contact distance, where the attractive forces are greater than the repulsive forces (*e.g.,* electrostatic repulsion), and thus the particles aggregate.

As we mentioned above, when individual colloidal particles remain dispersed, a colloidal system is considered ‘stable’. Particles do not aggregate when the forces keeping them apart are strong enough to prevent them from reaching the van der Waals contact distance (Lan et al., 2018). Charge stabilization is the primary mechanism of colloid stability in most aqueous systems. That is, the repulsive forces between particles, caused by electrostatic repulsion among particles with similar surface charges, prevent aggregation (Xu et al., 2018). As the diffuse layer becomes filled with counter-ions, the EDL is compressed. This effectively 'screens' the surface charge, permitting charged particles to approach as near as the van der Waals contact distance, which promotes aggregation (Xu et al., 2018). In water treatment systems, even the addition of counter-ions to neutralize surface charge is enough to induce aggregation and may be enough to clarify certain water systems.

## Coagulation and flocculation

### Principles of coagulation and flocculation

The technique of coagulation-flocculation involves the addition of a coagulant/flocculent to colloidal particles in water to destabilize them. As a result of this technique, the obtained aggregates can be effectively separated by sedimentation or filtration (Maddela et al., 2021). Flocculation is commonly used for the elimination of harmful substances from effluents. In flocculation, small particles aggregate and transform into flocs with suitable flocculants. Sodium dodecyl sulfate, poly ferric sulfate, and polyacrylamide are a few of the common flocculants commonly used in wastewater treatment. (Thakur, 2021). Coagulation and flocculation are advantageous treatment methods because they are cost-effective, easy to implement, and efficient at removing pollutants from water. The success and performance of the coagulation process primarily hinge on the selection and type of coagulant used (Benalia et al., 2024a; Saqib et al., 2024).

### Overview of coagulation mechanisms

It has been established through studies that the introduction of simple salts at high concentration into a stabilized colloidal dispersion results in the entry of their counter-ions into the diffuse double layer. This intrusion causes compression of the double layer, thereby lowering the long-range repulsion among colloids and enhancing aggregation due to van der Waals forces (Sreevidya et al., 2021). According to the Schulze-Hardy rule, this effect is increased proportionally to the counter ion charge. For instance, the relative effectiveness of Al^3+^, Mg^2+^, and Na^+^ in the coagulation of negatively charged colloids has been found to differ in the ratio 1000:30:1 (Sreevidya et al., 2021).

Generally, coagulation mechanisms are categorized based on the primary process that leads to the formation of aggregates. These mechanisms include: charge neutralization, sweep coagulation, the formation of inter-particle bridges, and double-layer compression. (Table 2) shows mechanisms for some natural coagulants used in water treatment.

**Table 2:** Classification of coagulants used in water purification and wastewater treatment

Coagulants	Subcategory	Examples
Chemical	Hydrolyzing metallic salts	Ferric chloride, ferric sulfate, magnesium chloride, and alum
	Pre-hydrolyzing metallic salts	Poly ferric chloride, poly ferric sulfate, poly aluminum chloride, poly aluminum sulfate, and poly ferric aluminum chloride
	Synthetic cationic polymers	Aminomethyl polyacrylamide, polyalkali, polyamine, polyethylene, and poly diallyldimethyl ammonium chloride
Natural	Plant-based	Moringa oleifera, Nirmali seeds, Tannin, Cactus, Potato starch, Tamarind seeds, Banana peel Guar gum, Gum Arabic, and Cactus latifaria extract
	Microorganism-based	Xanthan gum, Aspergillus sp., Enterobacter, Streptomonas
	Animal-based	Chitosan, Alginate, Chitin

#### Adsorption and charge neutralization

**Fig. 4:**
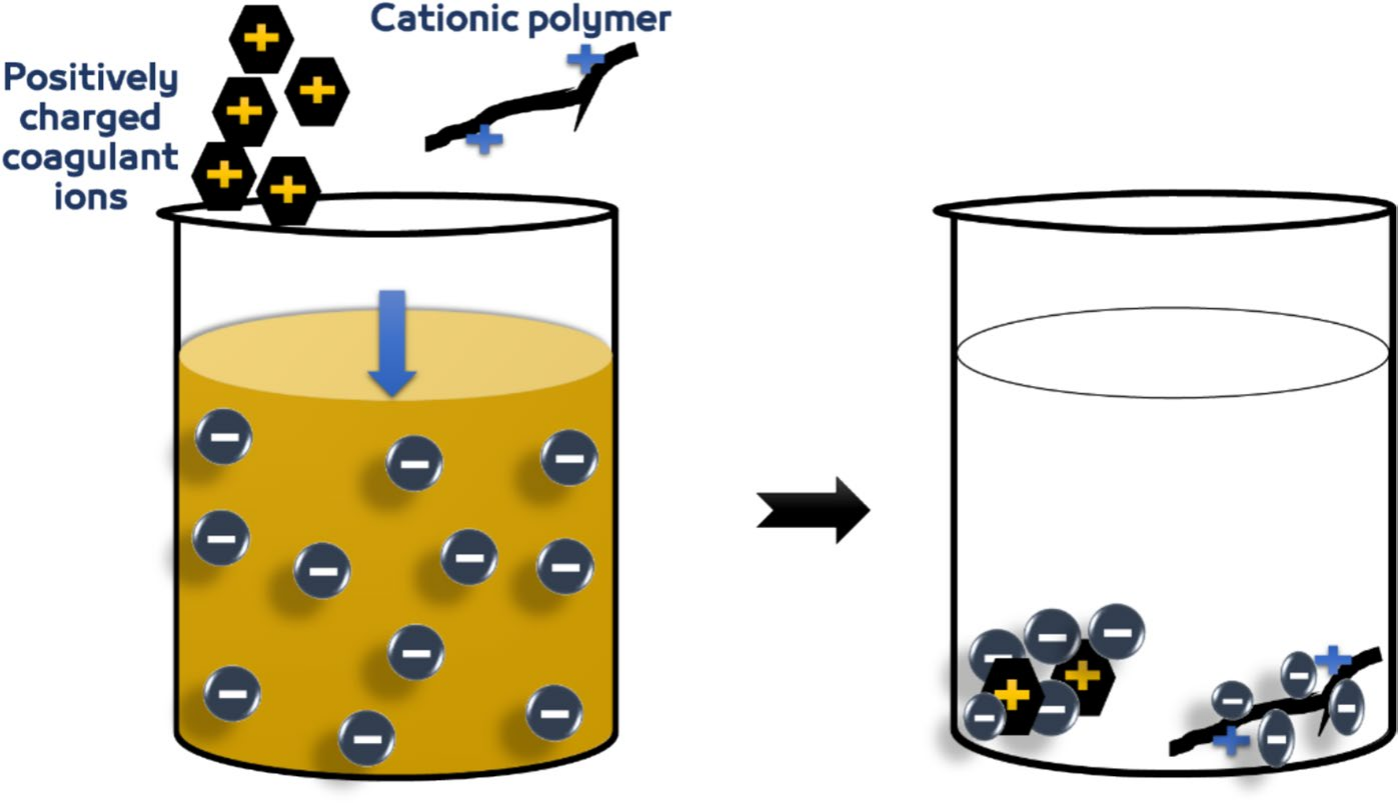
Charge neutralization coagulation mechanism

The process takes place in two phases. The first phase involves the chemical reaction between the coagulant and also the colloidal particles, reducing the repulsion forces between negatively charged colloidal particles. Reduction is triggered through the introduction of positive ions from the coagulant, which penetrate the diffuse double layers surrounding the particles before being adsorbed onto the surface of the particles. The particles therefore become denser and smaller in size, moving towards a smaller volume that allows the particles to be close to each other, the zeta potential is reduced to nearly zero net charge, as a result. The next step involves physically separating the formed aggregates, as the microflocs become denser and heavier, causing them to settle (Alnawajha et al., 2022). Figure 4 shows the adsorption and charge neutralization coagulation mechanism.

#### Sweep coagulation

**Fig. 5:**
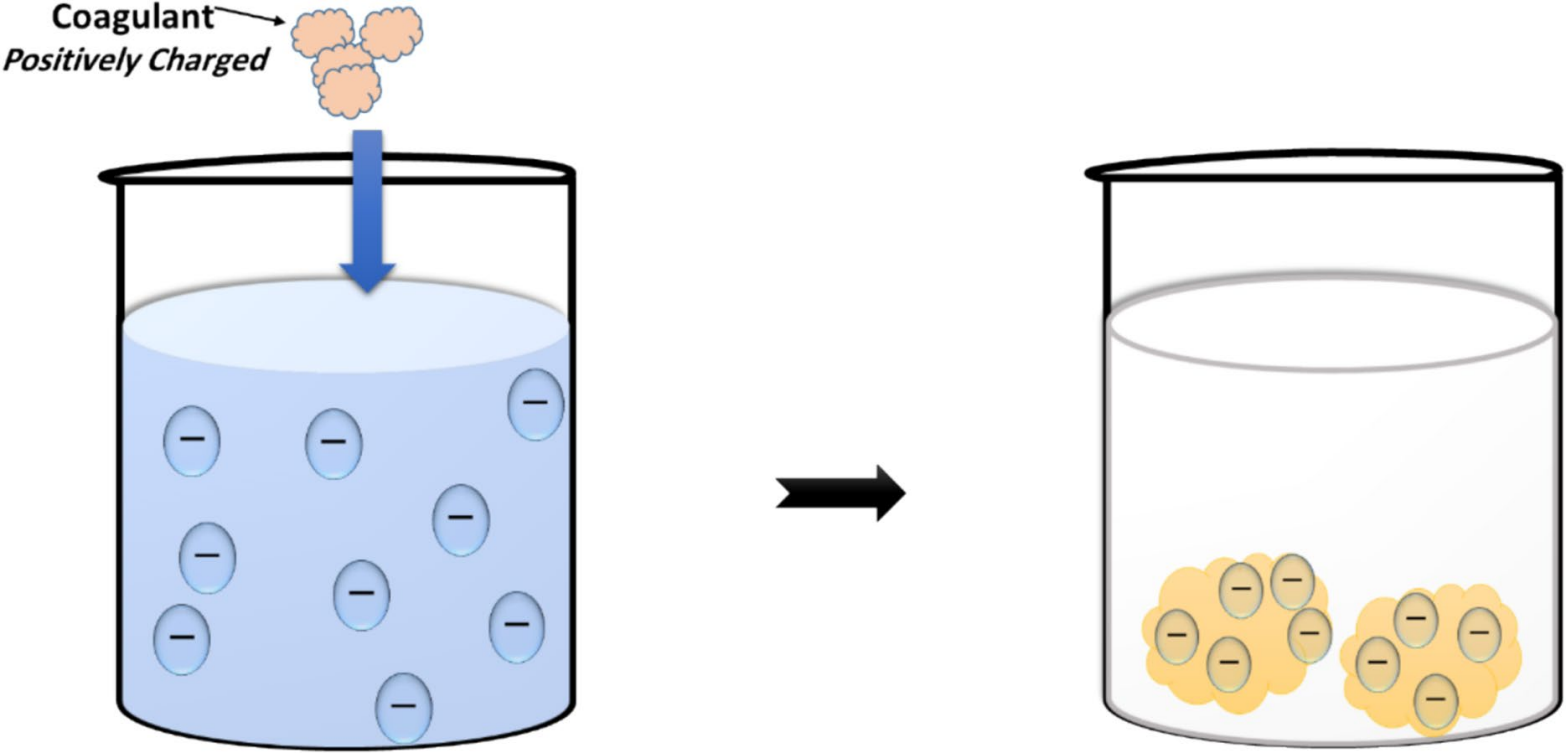
Sweep coagulation mechanism

Colloid entrapment is the process of adding relatively high concentrations of coagulants, usually aluminium or iron salts, that precipitate as hydrous metal oxides. The coagulant is added in an amount much larger than needed to neutralize the charge of the colloid. While some charge neutralization may take place, the majority of colloids are removed from the solution by becoming enmeshed within the settling hydrous oxide floc (Schalkwyk et al., 2016). Simply put, when a metal salt coagulant is added to water at a sufficiently high concentration to form amorphous metal hydroxide precipitates (M(OH)_x_), colloidal particles can become trapped within these precipitates (Fig. 5), this mechanism is called sweep coagulation (Suopajärvi, 2015).

#### Formation of inter-particle bridges

**Fig. 6:**
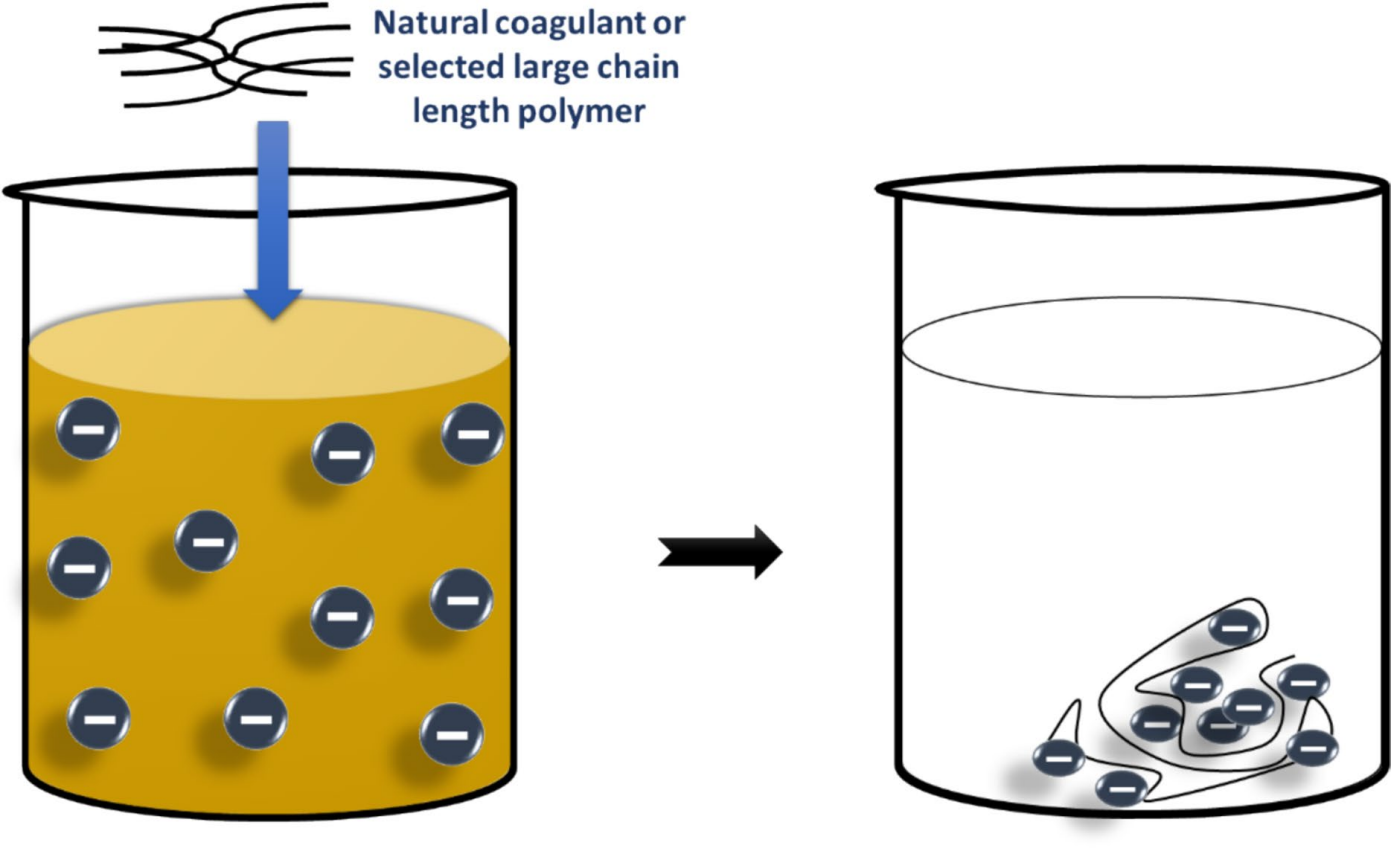
Inter-particle bridge mechanism

When the polyelectrolytes or polymers of large chain length reach out into a solution and they develop threads or fibers, then they create bridges that connect two or more particles of colloidal size (Fig. 6). Bridging is a very important function of the molecular weight of coagulant polymeric molecules (Mohd-Salleh et al., 2019). A low density of cationic and high molecular weight polymers works mainly by the bridging mechanism. Their long-chain molecules have many empty adsorption sites available, which facilitate efficiency (Alnawajha et al., 2022).

#### Double-layer compression

**Fig. 7:**
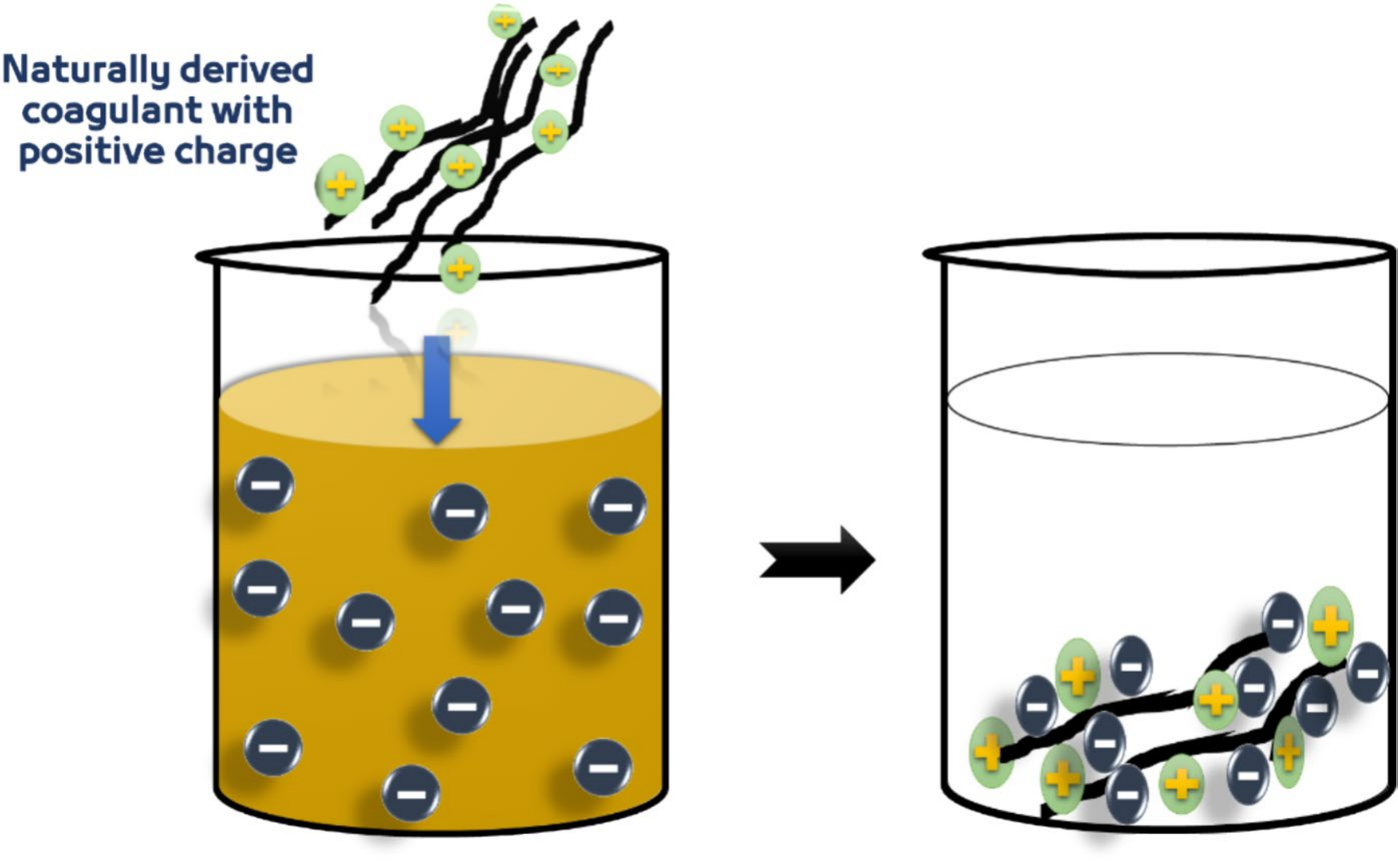
Double-layer compression mechanism

Negatively charged colloidal particles will attract oppositely charged ions to a tightly adsorbed layer near the particle, known as the stern layer. This causes a force of repulsion, and the equilibrium of this and the excess of the positively charged ions attracted to the negatively charged colloidal core results in the diffuse layer. The adjective “double-layer” is employed to refer to the two layers, *i.e.,* the stern layer and the diffuse layer, that are identified in the interfacial region around a colloidal particle (Zhao, 2020). The introduction of a positively charged coagulant to a colloidal solution results in double-layer compression of the colloidal particles. An introduction is brought about by forces of electrostatic attraction between opposite-charge colloids and ions (Fig. 7). Although double-layer compression is one of the most significant destabilization mechanisms in natural aquatic systems, it is not the predominant force causing colloid destabilization in water treatment processes (Ghernaout & Khums, 2020).

## Classification of coagulants

**Fig. 8:**
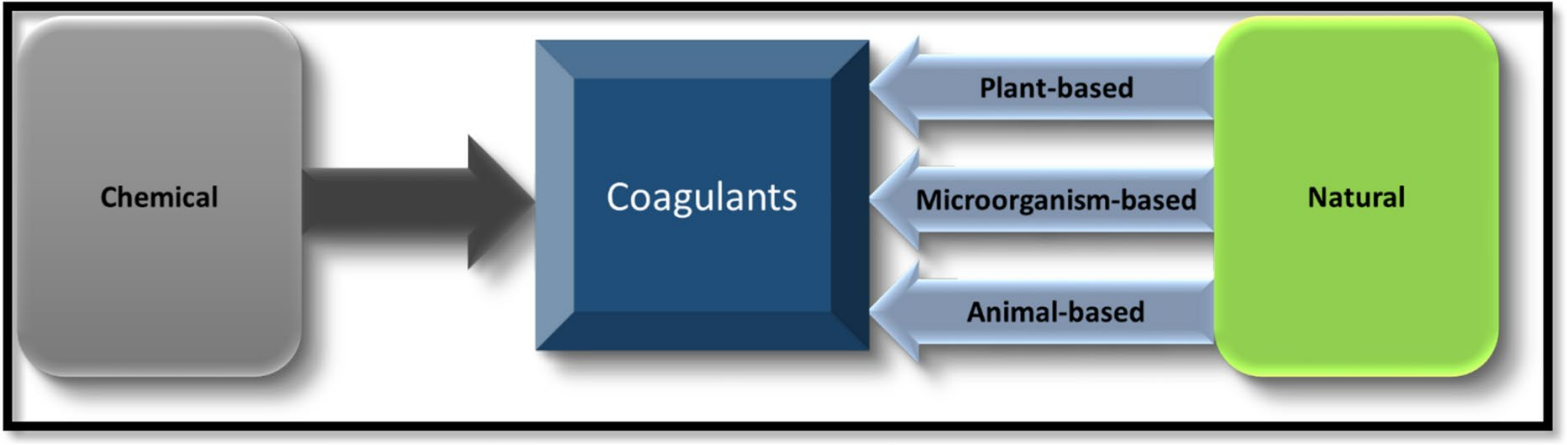
Categorization of coagulants applied in water and wastewater treatment processes

Various types of coagulants can be utilized for water treatment (Kweinor Tetteh et al., 2017). These coagulants may be chemical, or natural coagulants (Table 2 and Fig. 8) (El-taweel et al., 2023). Every coagulant has its distinct properties, with some containing positively charged ions that can trap the negatively charged organic matter in water responsible for turbidity (Tetteh & Rathilal, 2019). An overview of chemical, and natural coagulants, and coagulant aids is included in this section.

### Chemical coagulants

The use of chemical coagulants has been hugely controversial because of their toxic effect on living organisms. Chemical coagulants are generally categorized into three main groups: hydrolyzing metallic salts, pre-hydrolyzing metallic salts, and synthetic cationic polymers (Freitas, 2018). Chemical coagulants are frequently applied in wastewater treatment because they are economical, easy to manage, easy to store, and readily available. Al_2_(SO_4_)_3_, Fe_2_(SO_4_) _3_, AlCl_3_, and FeCl_3_ are the most commonly used coagulant salts. Although economic and operational factors favor the use of chemical coagulants, they lose based on their use according to green chemistry considerations and environmental concerns because most chemical coagulants add high amounts of aluminium to treated wastewater, an issue that poses serious concerns regarding the environment and human health (Freitas, 2018). According to Nimesha et al. (2022); Teixeira et al., 2017), the use of chemical coagulants poses significant risks to human health and surrounding ecosystems. For instance, aluminium residues from some of these coagulants can enter the human body, gathering in the brain, and have been associated with disorders like Alzheimer’s disease. Synthetic polymer coagulants often lead to dangerous byproducts like acrylamide, this toxic troublemaker is both carcinogenic and neurotoxic, posing serious health risks. Plus, these synthetic wonders boast low biodegradability. High levels of chemical coagulants, like that contain aluminium, lower water pH. These substances can also infiltrate food chains, accumulating silently over time. (Kurniawan et al., 2020). Toxic sludge tossed in the wrong way wreaks havoc on groundwater and soil. Think of aluminium and iron accumulating in our waters, creating a toxic cocktail. This contaminated concoction spells disaster for aquatic life and plant species alike. Therefore, we must harness natural coagulants for smarter water and wastewater treatment. It's time to turn the tide on pollution and protect our environment.

### Natural coagulants

Natural coagulants are riding a wave of popularity lately. Their myriad benefits shine through, addressing issues tied to traditional chemical coagulants. With nature as their ally, these coagulants offer effective solutions to persistent problems. Natural coagulants shine brightly in the coagulation process. Among them, plant extracts are stars—plentiful and devoid of harmful toxins (Kurniawan et al., 2023). These green warriors deliver reliable efficiency, promising safe and effective solutions (Awang & Aziz, 2012). Numerous studies investigate the use of natural coagulants, these coagulants come from animal, or agricultural waste materials. Noteworthy examples include moringa seeds, chitosan, and cassava peels (Mohd-Asharuddin, et al., 2017). Natural coagulants bring a breath of fresh air to wastewater treatment, these biodegradable wonders are gentle on both living organisms and the environment (Silva et al., 2025). Long before the rise of harsh chemical salts, they served as nature’s clarifiers, promoting purity with ease (Vieira et al., 2012). The lowering mass of natural polymers is the main reason for inefficient coagulation (Altaher et al., 2016). Natural coagulants are a blend of carbohydrates, proteins, and lipids. Their basic foundation consists of polysaccharide polymers and amino acids.

### Coagulant aids

Coagulant aids enhance the settling characteristics of coagulant flocs. Activated silica was a common coagulant aid used. Polyaluminium chloride (PAC) in combination with activated silica was used to enhance the coagulation of raw water from the Yellow River (YW) and the South-to-North Water Diversion (SNW) in Zhengzhou, China. Significantly, removal rates of turbidity as high as 96.5% were realized by applying a PAC dose of 35 mg/L in combination with an activated silica dose of 7 mg/L. Such treatment was given to a mixture of source waters, *i.e.,* YW and SNW, in a ratio of 2:1 (Lu et al., 2018). Some other coagulant aids that found application around this time were sodium alginates and certain soluble starch derivatives, which find use even to this day. Soluble starch products such as sago and tapioca were used for river water turbidity removal. Of particular interest is the fact that tapioca starch gave a 93.7% removal efficiency when used at a dosage of 1000 mg/L, while sago starch was more effective at 96.4% at its optimal dosage of 2000 mg/L (Zainol, 2020). Since these compounds had been well documented and already used in the food industry, they had the advantage of being recognized as safe for use in water purification. Later, the use of polyelectrolytes showed greater effectiveness. These materials now include a variety of synthetic substances, which are mostly long-chain organic chemicals that may be cationic, anionic, or non-ionic (Brandt et al., 2017).

## Natural coagulants: efficiency, and applications

### Composition and functional groups

The physico-chemical properties of natural coagulants play a fundamental role in their effectiveness during the coagulation–flocculation process. These properties, including surface charge, functional groups, solubility, …etc., directly influence their ability to destabilize colloidal particles and promote floc formation. For instance, the presence of hydroxyl, carboxyl, and amine groups enhances adsorption and charge neutralization, while factors such as pH, ionic strength, and dosage determine their coagulation performance. Characterizing these physico-chemical attributes is therefore essential for understanding the mechanisms of action of natural coagulants and for optimizing their application in water and wastewater treatment. Table 3 presents a comparison of the physico-chemical properties of selected natural coagulants.

**Table 3:** A comparative table of physico-chemical properties of some natural coagulants

Natural coagulant	Zeta potential	FTIR characteristics	Other properties	References
Walnut seed extract	− 24.73 mV at pH 10	FTIR shows presence of hydroxyl and carboxyl groups (proteins)	Porous, rough surface (SEM); contains C, N, O, Na, Mg, P, S, K, Ca, Fe, Cu; high protein content; lipid content reduced by 99% after extraction	Zedan et al. (2022)
Aqueous extracts of Musa paradisica (banana) peels and Dolichos lablab (Indian beans) seeds	The zeta potential of M. paradisica peel extract was consistently negative across all tested pH values (− 14.60 mV at pH 4.0 to − 37.60 mV at pH 11). In contrast, D. lablab seed extract showed a positive zeta potential at acidic pH (+ 2.39 mV at pH 3.0), which shifted sharply to a negative value (− 33.70 mV) at neutral pH (7.0)	FTIR spectroscopy of the extracts revealed that polymeric substances (carbohydrate and proteins) having functional groups –OH, C–N, C–C, –COOH, and N–H might be responsible for the coagulation activity	The scanning electron microscopy (SEM) analysis of the settled flocs revealed that more compact flocs formed using M. paradisica peels extract than those developed using D. lablab seeds extract	Daverey et al. (2019)
Moringa oleifera seeds	Typically cationic (positive charge)	FTIR shows protein-specific functional groups like –OH, –NH, COOH, C=O	Contains cationic polyelectrolytes effective in binding negatively charged particles; protein based; extraction and purification influence activity	Benalia et al. (2024b)
Cactus solid material (CSM)	the evolution of zeta potential displays a gradual decrease from − 7 mV at pH 3 to − 30 mV at pH 12, as does the synthetic water from − 10 to − 37 mV, respectively	FTIR of the CSM; a peak is found between 3200 and 3500 cm^−1^, which could be correlated with the existence of carboxylic acids. Band absorption between 2800 and 3000 cm^−1^ may be explained by the presence of CH_3_ and CH_3_–O. The vibrations of strips at 1620 and 1430 cm^−1^ indicate the existence of groups C=O and phenol groups	Removal efficacy depends on physicochemical properties including surface groups and charge	Bouaouine et al. (2018)
Lentil extract	Zeta potential close to zero in effective dose ranges	FTIR spectra reveal protein and polysaccharide groups	Used as effective coagulant in pulp & paper wastewater treatment, correlating zeta potential with COD and turbidity reduction	Ordaz-Díaz et al. (2017)
Crescentia cujete fruit shell	− 3.42 mV	The FT-IR analysis revealed the presence of hydroxyl (O–H), carbonyl (C––O) and alkyl (C––C, C=C) functional groups	This coagulant has significant potential as an alternative to inorganic coagulants for the removal of water turbidity, and further research can be done to explore its use on a commercial scale	Boakye et al. (2024)
Brown, green, and red lentil (lens culinaris) extracts	The triplicates zeta potential results of brown, green, and red lentils were − 3.74 mV, − 2.91 mV, and − 5.91 mV, respectively, which revealed the anionic properties of the lentil extracts	The spectrum observed for all three lentils extracts were similar. The strong peak observed at 3400–3294 cm^−1^ was attribute to the OH functional group and the O–H stretching of the polymeric compound. Besides, the peak at 2929–2928 cm^−1^ was recognized as C–H groups. Both O–H and C–H functional groups could be attributed to the protein content found in the lentil extracts	Based on SEM images, all three lentil extracts exhibited same rough surface with pores and obvious surface abrasions	Chua et al. (2019)

### Natural coagulants: mechanisms and efficiency comparison

The efficiency of natural coagulants in treating water is reliant on three components: the nature of the coagulant, the nature of the water to be treated, and the nature of the mixing (Ang et al., 2020). Surface charge is the controlling parameter; positively charged coagulants will perform optimally with negatively charged particles, and vice versa. This charge behavior is brought about by functional groups, while molecular weight contributes significantly to particle bridging—high molecular weight polymers promote the formation of stronger, more stable flocs (Ang et al., 2020). The level of mixing intensity also influences coagulation, with the formation of microfloc being encouraged by fast mixing and subsequent slow mixing aiding their growth into larger aggregates (Kurniawan et al., 2020).

Natural coagulants are composed predominantly of polysaccharides and proteins, and their coagulation is governed by two mechanisms in the main: charge neutralization and polymer bridging. In the latter, the adsorption of long-chain polymers on colloidal particles takes place, with loops and tails stretching outwards to bind onto other particles and create larger flocs. Charge neutralization, on the other hand, is induced through electrostatic attractions, where oppositely charged surface patches on particles promote aggregation (Table 4). Polyelectrolytes, more often polycations, are typically involved in this mechanism, reducing zeta potential towards neutrality (Amran et al., 2018). Natural coagulants in general have diverse modes of action, and their effectiveness is strongly reliant on coagulant chemistry, molecular structure, and process conditions (Nimesha et al., 2022).

**Table 4:** Natural coagulants: modes of action and applications in water treatment

Natural coagulants	Coagulation mechanism	References
Mustard seed	Adsorption and charge neutralization	Nimesha et al., (Al-Sahari et al. (2020); Diver et al. (2023); Li et al. (2013); Nweke et al. (2021); Shammas et al. (2021; 2022))
Nirmali	Interparticle bridging	
Groundnut shell	Enmeshment in a precipitate	
Banana pith powder– banana pith juice	Charge neutralization combined with low bridging	
Cactus mucilage	Adsorption and bridging coagulation method	
Chitosan	Charge neutralization and bridging	

The potential of different plant-based materials to serve as natural coagulants is summarized in Table 5.

**Table 5:** An overview of the removal efficiencies of some natural coagulants in water and wastewater treatment

Natural coagulant	Wastewater source	Dosage	pH	Parameters reduced/pollutant	Removal Efficiency (%)	References
Fenugreek seeds	Synthetic turbid water	10 ml per 500 ml turbid water	8	Turbidity	98	ELsayed et al. (2021)
*Hibiscus sabdariffa* (roselle seed extract)	Glove manufacturing wastewater	60 mg/L	10 ≤	Turbidity	87	Saharudin, N.F.A.b. and R. Nithyanandam (2014)
*Corchuros olitorius L*.(50%) + Alum (50%)	The domestic wastewater	50 mg/L	7	Turbidity	80.27	Lacang and Ligaray (2023)
*Moringa oleifera* (drumstick Tree)	Laundry wastewater	120 mg/L	5.7	COD Turbidity	43 84	Al-Gheethi and et al. (2017)
*Chrysopogon zizanioides*	Turbid lake water (Puzhal Lake)	100 g/L	6.7	Turbidity	77.24,	Asaithambi et al. 2024)
*Hemidesmus indicus*	Turbid lake water (Puzhal Lake)	100 g/L	6.5	Turbidity	80.23%	Asaithambi et al. (2024)
*Opuntia ficus indica* (cactus species)	Textile wastewater	40 mg/L	7.25	COD Color	89 99	Bouatay and Mhenni (2014)
*Citrullus lanatus* (seeds of water melon)	Tannery wastewater	2000 mg/L	7.1	Turbidity BOD COD TSS	87 55 50 69	Sathish et al. (2018)
Aloe vera			12	Turbidity TSS Organic matter Aromatic matter	99.13 94.01 56.26 88.34	Benalia et al. (2024c)
*Strychnos potatorum* (nirmali seeds)	Laundry wastewater	8000 mg/L	-	Turbidity TSS	96 76	Mohan (2014)
*Musa paradisica* (banana) peels		0.6 mL/L	11	Turbidity	98.14	Daverey et al. (2019)
*Ocimum bsailicum* (basil)	Textile wastewater	1600 mg/L	8.5	COD Color Turbidity	62 69 95	Shamsnejati et al. (2015)
*Corchorus Olitorius L* (jute mallow)	Agricultural wastewater	3 mg/L	7.5	(Total organic carbon)	100%	Altaher et al. 2016)
*Dolichos lablab* (Indian beans) seeds		0.6 mL/L	11	Turbidity	(98.84)	Daverey et al. (2019)
*Moringa oleifera* (drumstick tree)	sewage, gray water (water from sinks and showers)		7 ≤	Turbidity COD BOD TSS TDS Hardness	61 65 55 69 68 25	Kumar Kaushal and Goyal (2019)

### Coagulation kinetics

The kinetics of coagulation and flocculation processes in municipal water treatment plants is essential to maximizing treatment efficacy. These processes have time-dependent physicochemical interactions between destabilized particles, coagulants, and flocculants that induce the formation of flocs, which can be settled or filtered out. The rate of such interactions matters since it determines not only the removal efficiency of pollutants but also the overall operating and chemical demand throughput of the treatment system (AFOLABI, M., et al., 2020).Table 6 highlights the coagulation kinetics associated with selected natural coagulants.

**Table 6:** Coagulation kinetics of selected natural coagulants

Natural coagulant	Coagulation kinetics	References
Brachystegia eurycoma coagulant (BEC) and Vigna subterranean coagulant (VSC)	The kinetic data obeyed the second-order reaction. Also, the correlation coefficient (R^2^) demonstrates good agreement that implies that the studied kinetic data is significant	Obiora-Okafo et al. (2022)
Ferromagnetite (F), alum (A), and eggshells (E) and their hybrids (FA, FE, and FEA)	It is obtained that the coefficient of determination (R^2^) for the single coagulant dosage was favored by the second-order kinetic model. Whereas the first-order kinetic model showed a good fit for the combined coagulant dosage	Precious Sibiya et al. (2021)
Garcinia kola (bitter cola) seeds	The kinetic data obeyed the second-order kinetic model	Igwegbe et al. (2021)
Hibiscus esculentus L, (okra) Detarium microcarpum, (sweet dater) Xanthosoma (cocoyam) and Crassostrea Virginica, (oyster shell)	the data fitted best into the second-order coagulation-flocculation equation	Okolo et al. (2021)
Moringa oleifera seeds	The value demonstrated that the observed data fit with the first-order kinetics model	Varsani et al. (2022)
Groundnut shell	The experimental data fitted Von-Smoluchowski’s second-order coagulation kinetic at 298 K than at 308 K and 318 K	Nweke et al. (2021)

### Field and pilot-scale applications

Pilot-scale studies play a fundamental role in bridging laboratory studies and full-scale implementation. While laboratory-based experiments provide controlled conditions for evaluating coagulant performance, they often fail to capture the operational complexities of real water treatment systems. Pilot-scale trials allow researchers to evaluate the scalability of natural coagulants under variable water quality conditions, continuous flow regimes, and realistic hydraulic loading rates. Moreover, such studies provide critical data on process optimization, economic feasibility, and potential operational problems, thereby reducing risks associated with large-scale implementation and easing the way for industrial application. A pilot-scale experiment was carried out to replicate full-scale treatment conditions using the C. fistula–CoFe₂O₄ composite. Under these conditions, a colour removal efficiency of 94.0% was attained when treating 30 L of wastewater with a coagulant dose of 12.3 g L⁻^1^ (Vo et al., 2024). In another study, the ideal conditions established from jar tests were applied to a pilot-scale coagulation–flocculation–sedimentation system flowing at 150 L/h. With Aloe vera–based bio-coagulants, the process attained removal efficiencies of 98.45% for turbidity and 92.29% for total suspended solids (TSS) (Benalia et al., 2024c). A study explored the use of pine cones, an innovative and largely untapped waste resource, as a bio-coagulant for application in wastewater treatment plants, the optimized parameters were subsequently tested at pilot scale, yielding removal rates of 97.77% for turbidity, 71.35% for COD, and 88.6% for phosphate. These results demonstrate that pine cones offer an efficient, economical, and environmentally sustainable option for water treatment plants (Baatache et al., 2024a).

### Potential toxicological effects of natural coagulants on humans and the environment

Although bio-coagulants are often regarded as safe because of their natural origin, the toxicological profile of organic coagulants and their broader impacts on living systems are not yet fully understood. This issue becomes more critical when natural coagulants are chemically modified, as the grafting of functional groups using acids or bases may alter their safety. Therefore, comprehensive studies addressing the potential effects of natural coagulants on both human health and the environment are necessary to ensure their reliable and safe application in water treatment (Benalia et al., 2024b).

### Insights into sludge generated from eco-friendly coagulants

In the process of wastewater treatment, coagulation and flocculation generate a by-product called coagulant sludge. It contains residual suspended material, entrapped impurities, and residues of the coagulant itself. Due to its potential environmental hazard, the management and disposal of this sludge are of significant concern. If not disposed of correctly, it can become a secondary source of pollution through releasing entrained pollutants into nearby soils and water bodies, which can have negative impacts on ecosystems and human health (Badawi et al., 2023).The application of biocoagulants generates sludge with high biodegradability; thus, The use of sludge is actually possible. Moreover, the further processing of sludge through anaerobic digestion is suitable and gives useful gas as a byproduct (Lichtfouse et al., 2019). Biocoagulants are nontoxic no effects on the anaerobic digestion microorganisms and therefore will not disrupt their activity (Hamawand et al., 2017). The trend now also shows the promise of the use of generated sludge from wastewater treatment as a fertilizer/soil conditioner. The wastewater treatment sludge in agricultural industries, including aquaculture, the palm oil industry, sago farming, and coffee, generally is rich in organic contents and nutrients (Kurniawan et al., 2020; Said et al., 2020). Sludge organic content can be further composted to be used as a soil conditioner, while the nutrients therein will act as a soil Fertilizer. Recovered solids from municipal wastewater treatment plants can have up to 15% Phosphorus (You et al., 2019) can find uses in the field of agriculture. Also, in connection to the previous statement, Kominko et al. (Kominko et al., 2017) reported the possibility of producing an organo-mineral fertilizer based on domestic wastewater. The use of the generated sludge will not only minimize the possible damage to the environment but could also benefit the agricultural sector Through the incorporation of such strategies and the pursuit of valuable reuse possibilities, coagulant sludge management can proceed in a more sustainable and resource-effective direction.

## Life cycle and sustainability perspectives

### Life cycle assessment

Life Cycle Assessment (LCA) is a standardized methodology used to evaluate the environmental impacts of a product or process throughout its entire life cycle, from raw material extraction to end-of-life, thus providing a holistic measure of sustainability. Several studies have applied LCA to natural coagulants. For example, an LCA on coagulants derived from common bean seeds (Phaseolus vulgaris) showed that electricity consumption during the spray-drying stage was the dominant contributor, accounting for nearly 90% of the overall environmental impact (Radovic et al., 2023). In another study, the large-scale production (> 500 tons/year) of a tannin-based agent (TBA) from Acacia spp. was assessed using openLCA (version 2.0.0) under a cradle-to-gate approach. The results, which also compared TBA production with aluminum sulfate as a conventional coagulant, highlighted the importance of such assessments in identifying the most impactful categories and guiding improvements toward more sustainable production (Santos et al., 2024).

### Technology readiness levels of natural coagulants for water and wastewater treatment

Currently, the integration of natural coagulants for water treatment remains at mid-range Technology Readiness Level (TRL), predominantly within TRL 3–4. For instance, jar-test studies using chitosan demonstrate its effectiveness in turbidity removal under laboratory conditions, but with operational and regulatory constraints remaining (Pontius, 2016) Moreover, practical considerations such as inconsistent quality, packaging logistics, and the absence of National Sanitation Foundation (NSF) Standard 60 certification for chitosan further limit its industrial readiness (Shipper-focused analysis). Similarly, recent experimental work using aloe vera seed extracts shows promising turbidity removal performance at bench-scale level, reinforcing its current placement at TRL 3 (Ahmed, 2023). Broader reviews of plant-based coagulants confirm that while environmentally appealing, most have not yet advanced beyond laboratory or small pilot studies, with key challenges—including storage stability, raw-material consistency, and regulatory clarification-hindering scale-up (Koul et al., 2022).

### Shelf life, storage stability, and degradation profile of natural coagulants

Shelf life and storage stability of natural coagulants, including Moringa oleifera seed extracts, are very important parameters in industrial practice. Generally, raw aqueous extracts show significant loss of coagulation activity in a period of days at room temperatures; for instance, turbidity removal efficiencies undergo significant decline after just 3 days at around 28 °C, with effectiveness coming to a near halt between days 5 and 7 for medium- to low-turbidity water sources. Nevertheless, proper processing methods, such as freeze-drying, combined with proper packaging procedures, can significantly enhance shelf life: freeze-dried seed materials retain high coagulation activity up to 11 months under a variety of storage conditions, whereas non-freeze-dried seeds stored in sealed or vacuum-packed containers at 4 °C show better retention of effectiveness than seeds stored at room temperature (Katayon et al., 2006). Moreover, dried seeds stored in dry jars at room temperature for a number of years, reaching up to around 4.5 years, did not exhibit any statistically significant decrease in coagulant activity during jar testing (Diaz et al., 2018).

### The cost

According to (Ho et al., 2025; Nimesha et al., 2022) natural coagulants are characterized by their low cost, making them an attractive alternative to conventional chemical coagulants. Their affordability, coupled with biodegradability and low environmental impact, positions them as a sustainable and economically viable solution for water and wastewater treatment. A comparative assessment of various coagulants was made based on various parameters, such as cost. Economic analysis indicated that, under optimal conditions, the harvesting of 1 metric ton (MT) of wet algal biomass would cost approximately $0.28 when alum was used. Surprisingly, when a natural coagulant was used, the cost was drastically reduced to only $0.037 per MT. This low cost is mainly due to the electricity used in the stirring process. The findings accentuate the improved cost-effectiveness of natural coagulants over their chemical alternatives, especially amidst the pursuit of environmentally friendly and economically viable water treatment solutions (Behera & Balasubramanian, 2019).

Another research article presented in 2023 assessed alum (an inorganic coagulant) as compared to neem leaf extract (a plant-based coagulant) to treat aquaculture wastewater based on efficiency. The study reported that even though alum performed superior removal efficiencies toward total suspended solids, turbidity, and color, neem extract could obtain optimum performance at a lower dosage. Nevertheless, the cost–benefit analysis showed that both coagulants produced negative net profits, demonstrating that neither option was economically favorable under the conditions of the study. Nonetheless, the utilization of neim coagulant provided advantages for water recycling and sludge reuse, highlighting potential non-financial benefits (Kurniawan et al., 2023).

### Regulatory guidelines discussion and academic references for natural coagulants in drinking water

Natural (bio-based) coagulants such as Moringa oleifera extracts, tannins, and chitosan have attracted increasing attention as sustainable alternatives to conventional coagulants. However, their application in drinking-water treatment must comply with existing regulatory frameworks that govern all water treatment chemicals. In the United States, the Safe Drinking Water Act (SDWA) regulates treatment chemicals through treatment technique (TT) limits on monomer impurities, such as acrylamide (≤ 0.05% at a dose of 1 mg/L) and epichlorohydrin (≤ 0.01% at 20 mg/L). The U.S. Environmental Protection Agency (USEPA) also enforces a Secondary Maximum Contaminant Level (SMCL) for aluminum in the range of 0.05–0.2 mg/L, which utilities often adopt as an operational target (USEPA, 2025). Compliance with the NSF/ANSI/CAN-60 certification standard is further required for all drinking-water treatment chemicals, including bio-derived products. At the international level, the European Union Drinking Water Directive (2020/2184) establishes a parametric value of 200 µg/L for aluminum and requires risk-based assessment of all treatment chemicals to ensure their purity and absence of harmful by-products. Similarly, the World Health Organization does not set a health-based guideline value for aluminum but recommends operational targets, with well-optimized plants typically achieving residuals ≤ 0.1 mg/L. These regulatory thresholds underscore the need for thorough characterization of natural coagulants to verify chemical purity and establish safe dosing ranges. Academic studies support the feasibility of bio-coagulants within such frameworks. For example, Moringa oleifera seed extracts have been shown to effectively reduce turbidity and organic load in water, aligning with regulatory requirements for clarity and safety (Ng & Elshikh, 2021). Likewise, chitosan has demonstrated significant coagulation efficiency, enabling treated waters to meet USEPA turbidity standards (≤ 0.3 NTU) and showing promise as a safe and effective natural coagulant (Pontius, 2016). Collectively, these findings highlight the dual necessity of innovation in natural coagulants and strict adherence to established regulatory and certification guidelines to ensure their safe implementation in drinking-water treatment systems (Coleman et al., 2024).

## Factors influencing the coagulation flocculation process

### Effect of solution pH on coagulation

The pH value is a controlling factor in determining the charge properties of both coagulant and pollutant particles and is, therefore, a significant variable in optimizing the performance of both natural and mineral coagulants (Baatache et al., 2024b). Coagulants will achieve their optimum efficiency in a specific pH range, in which they possess their optimum stability and charge. For instance, metal coagulants like alum (aluminium sulfate) and ferric chloride exhibit their optimum activity in a weakly acidic to neutral pH range, typically between 6 and 8. Under this pH condition, the coagulant molecules experience hydrolysis and subsequent precipitation of positively charged metal hydroxide species that exhibit high efficiency in neutralizing negatively charged leachate particles. At low pH levels, the effective elimination of leachate’s chemical oxygen demand (COD) is largely attributed to charge neutralization. At higher pH levels, though, sweep-flocculation becomes the predominant mechanism with greater effectiveness in color and suspended particulates' removal. These differences in mechanisms highlight the importance of pH control in optimizing coagulation processes for specific treatment goals, such as COD reduction or turbidity and color removal (Li et al., 2020). Several researchers have investigated how pH can influence coagulants' performance. Benalia et al. (Benalia et al., 2024c), carried out a study on the application of the bio-coagulant (Aloe vera) for wastewater treatment and assessed its efficiency in the range of initial pH 2 to 12. The findings indicate that the maximum removal of turbidity (99.13%), total suspended solids (TSS) (94.01%), organic matter (56.26%), and aromatic matter (88.34%) occurred at pH 12.

Another investigation has pointed out the significance of coagulant dose in establishing the optimum pH for the coagulation-flocculation process. This investigation has illustrated that, for natural organic matter, acidic pH is effective for its removal via a charge neutralization mechanism (Saritha et al., 2017). Banana peels (Musa paradisiaca) can generate stable coagulants that remain effective across a broad pH range (3–12), whereas extracts from Indian bean (Dolichos lablab) seeds tend to be more pH-sensitive (Daverey et al., 2019). Coagulants that remain stable across a broad pH range are especially beneficial, as they sustain consistent efficiency throughout the coagulation process, leading to an effective reduction of water turbidity (Daverey et al., 2019).

### Effect of coagulant dosage

Coagulant dosage is a significant aspect of the coagulation process as it directly affects the coagulant agent and water colloidal particles' interaction (Benalia et al., 2024c; Mohd-Asharuddin, 2018), as well as playing a major part in coagula formation. According to this study (Zhang et al., 2024), when no coagulant was added (0 mg/L dose), the average floc size measured was 24.15 µm, which is very low, this indicates that, without coagulant, the particles in water are dispersed and not well aggregated. As coagulant dosage increased, the average size of the formed flocs showed a steady rise from 24.15 µm without coagulant to 134.22 µm, 195.5 µm, and eventually 254.45 µm at higher dosages. Some flocs are seen to become considerably larger when the coagulant dosage is higher than 90 mg/L. Gao et al. (Gao et al., 2019) showed that if the dosage of the coagulant is more than a certain value, a marginal reduction in settling velocity can occur since residual fine particles, which are not fully coagulated, require more time for aggregation. However, it can be seen that within a certain range of dosage of the coagulant, coagulation greatly enhances floc size and enhances sedimentation rates. This enhanced sedimentation is beneficial in realizing more effective solid–liquid separation since larger and heavier flocs settle faster and thoroughly. Optimizing the coagulant dose within this range is thus essential to realize maximum treatment efficiency in water and wastewater treatment (Zhang et al., 2024).

### Effect of coagulant type

The choice of the coagulant is extremely broad and critical in the event of coagulation-flocculation separation of a liquid/solid mixture since it exerts a great impact on the separation processes (filtration, decantation, and flotation) and reduces the amount of sludge generated. The nature of the coagulant, whether natural or synthetic, affects the coagulation and flocculation processes in water treatment. The coagulants may be used by themselves or in conjunction with other agents to promote the overall effectiveness of coagulation and flocculation (Saritha et al., 2017). Natural and chemical coagulants differ in the stability of suspended particles and in their ability to create flocs, which have varied effects on floc size and strength. For example, chemical coagulants create weak and smaller flocs, while organic coagulants assist in creating stronger and larger flocs (Putra & Fitria, 2023). However, it is important to consider natural coagulants' chemical structure (*e.g.,* fruits and leaves) in trying to optimize the processes involved. This entails observation of the type of coagulating agent—*e.g.,* proteins or tannins—and the routes by which the agents interact with the pollutants (Benalia et al., 2024a).

### Effect of temperature

Temperature is also an important parameter in the coagulation-flocculation process (Ezemagu et al., 2021), at low temperatures, the high viscosity of water hinders the settling of flocs and lowers the solubility of the coagulants. According to Feng et al., lower temperatures significantly hinder the kinetics of floc aggregation (Benalia et al., 2024a; Xiao et al., 2009). Research has also examined the influence of temperature on the optimal coagulation pH range, with the suggestion that an increase in temperature needs a modest adjustment in coagulation pH. This correlation is linked to the stoichiometry of coagulation that shows temperature dependency as well. Research findings presented by Guan et al. depicted that elevated water temperatures increased the efficiency of turbidity removal, with maximum reductions of 66.20% at 10 °C and 76.06% at 18 °C over a pH range of 6–9 (Guan et al., 2011).

### Effect of initial turbidity

Initial turbidity is a significant parameter that influences the coagulation process. At extremely high turbidity (above 100 NTU), the coagulant dose needed is typically less than that for low-turbidity water (5–10 NTU). Water with high turbidity contains a greater concentration of suspended particles, which increases the frequency of particle collisions and thereby the coagulation efficiency (Wang & Chen, 2022), greater collisions of particles lead to the formation of larger and more robust flocs that will settle rapidly, flocculation and coagulation are favored and therefore lower coagulant dosages are applied. Low-turbidity water also requires a larger coagulant dosage because the collision frequency between coagulants and impurities is significantly reduced. Having fewer suspended particles in the water, there is a decreased opportunity for effective interaction, and more coagulants should be applied to enable aggregation and form flocs. Consequently, small flocs with slow-settling behavior are produced. Furthermore, low initial turbidity typically results in a flake-like morphology of lower density that takes longer to settle (Alazaiza et al., 2022).

Different studies have investigated the impact of initial turbidity of water on the efficiency of coagulation. For instance, Odiyo et al. (Odiyo et al., 2017) determined that coagulation efficiency was maximum in very turbid water (380 NTU) where 71% removal was attained. However, as initial turbidity decreased (65 NTU), the coagulation efficiency decreased with the highest turbidity removal of only 34.3% (Odiyo et al., 2017). In a different study conducted by Nkurunziza et al*.,* (Janna, 2016), Moringa oleifera was less effective as a coagulant in low-turbidity water (50 NTU) compared to high-turbidity water (450 NTU). Turbidity removal efficiencies were 88.06% and 99.80% for 50 NTU and 450 NTU, respectively. The optimum coagulant dosages were 150 mg/L and 125 mg/L for 50 NTU and 450 NTU, respectively, indicating that the dose needed is decreasing with an increase in turbidity. According to Janna (2016), turbid water is categorized into: low and high based on their values (in NTU); water is said to have high turbidity if its turbidity is between 90 and 120 NTU, while that of low turbidity is between 20 and 35 NTU. Alternatively, turbidity less than 20–35 NTU defines low turbidity, while greater values of over 90–120 NTU define high turbidity (Janna, 2016). Asrafuzzaman et al. (Asrafuzzaman et al., 2011) investigated the application of bio-coagulants, *i.e.,* Moringa oleifera, Cicer arietinum, and Dolichos lablab, for turbidity removal at various levels: (i) low (25–35 NTU), (ii) medium (40–50 NTU), and (iii) high (> 50 NTU). It was observed that these bio-coagulants were more effective in treating highly turbid water compared to their efficiency in slightly or moderately turbid water. For example, the application of Cicer arietinum as a coagulant resulted in 71.29% turbidity removal efficiency in low-turbidity water, 81.63% in moderately turbid water, and 95.89% in highly turbid water.

### Effect of the mixing speed

The mixing speed constitutes a key operating parameter affecting the efficiency of coagulation. Intensive mixing is needed at the point of coagulant addition to achieve uniform dispersal and also to promote the destabilization of suspended particles. Gentle mixing is required, however, to increase particle interaction to aid the development of larger and stronger flocs (Gautam & Saini, 2020). Both these mixing velocities govern the overall coagulation process, as the success of this process is reliant on both the mixing velocity and time. Low mixing velocity or time may inhibit effective particle aggregation, whereas too high mixing can cause shear and breakage of flocs, thereby lowering the overall efficiency of coagulation (Alazaiza et al., 2022).

### Effect of settling time

The optimal settling time is the time at which turbidity and/or TSS are minimum or is constant (Padhiyar et al., 2020). Bhatia et al. also investigated the settling time effect (30–150 min) on coagulation efficiency using Moringa oleifera as a sole coagulant. The result indicated that optimum efficiency for suspended solids removal, attained at 90.09%, occurred at 114 min of settling time (Bhatia et al., 2007). A further study determined the optimal settling time based on the pair of wastewater coagulants and the minimum residual turbidity achieved at the optimized dose. The impact of sedimentation was investigated in the range of 30–360 min on residual turbidity. Optimum settling times for turbidity removal were determined to be 240 min for Moringa oleifera-treated dye-loaded wastewater, 60 min for Moringa oleifera-treated starch-loaded wastewater, and 120 min for Moringa oleifera-treated textile industrial wastewater (Padhiyar et al., 2020).

## Limitations and future research

To truly harness the potential of natural coagulants, a deeper, more detailed comparison between natural and chemical coagulants is crucial. While chemical coagulants like alum and ferric chloride have long been recognized for their ability to efficiently remove a wide range of contaminants, they come with their own set of challenges. These include the generation of large volumes of sludge, health risks, and significant environmental concerns. In contrast, natural coagulants often demonstrate comparable—and in some cases, superior—turbidity removal efficiency. However, their performance can fluctuate based on factors like pH and temperature, which are less predictable in real-world conditions. Moreover, sourcing, processing, and standardizing natural coagulants continue to be areas where much more research is needed. It’s clear that the long-term environmental impacts of both coagulants—focusing on biodegradability, toxicity, and sludge management—are essential areas for future investigation.

Though impressive strides have been made in the study of natural coagulants, several key challenges remain. The effectiveness of these coagulants can vary greatly, depending on the source material and its geographical origin. Take Moringa oleifera, for example: the coagulating power of its seeds can be influenced by the climate and soil conditions where it’s grown. This geographical variability introduces an element of uncertainty that needs to be addressed. Additionally, while laboratory studies have demonstrated the excellent performance of natural coagulants, their efficacy in large-scale, real-world water treatment systems remains an area requiring much further exploration.

Looking ahead, the next frontier of research should focus on standardizing the extraction and processing methods of these natural coagulants. Consistency is key, and finding ways to optimize these processes will be crucial for large-scale application. Moreover, the economic feasibility of natural coagulants—particularly their production costs, scalability, and long-term sustainability—needs to be better understood. While natural coagulants offer a greener alternative to chemicals, there’s still much to explore in terms of their practical and cost-effective implementation.

Scaling up the use of natural coagulants to meet the demands of industrial and municipal water treatment brings its own set of challenges. Variations in coagulant quality and availability, driven by seasonal changes and geographic factors, could lead to inconsistencies in performance. Moreover, questions surrounding the cost-effectiveness of large-scale production processes, such as extraction and refinement methods, remain unresolved. To truly realize the potential of natural coagulants in the industry, it’s imperative to develop standardized protocols that guarantee consistent quality and dosage.

The path forward lies in ongoing research into optimizing extraction and processing methods. The development of industry-wide standards and guidelines will be key to the successful integration of natural coagulants into large-scale water treatment operations.

Looking further into the future, there’s exciting potential in hybrid systems that combine the strengths of both natural and chemical coagulants. These innovative solutions could bridge the gap between performance and sustainability, ensuring that we can harness the best of both worlds—delivering superior treatment while minimizing environmental impact. The future of water treatment is not just about technology; it’s about marrying the power of nature with cutting-edge innovation.

## Conclusion

The growing concerns about water scarcity and pollution highlight the urgent need for sustainable water treatment solutions. While traditional chemical coagulants, such as alum and ferric chloride, are effective in removing contaminants, they generate toxic sludge, raise health concerns, and contribute to environmental pollution. In contrast, natural coagulants, derived from renewable resources like plants and microorganisms, offer an eco-friendly alternative, with benefits such as biodegradability, lower toxicity, and reduced sludge production.

Despite their potential, challenges remain in scaling natural coagulants for large-scale industrial and municipal applications. Performance can vary based on factors like source material quality, pH, and temperature, and there is a need for standardized extraction and processing methods to ensure consistent quality. Furthermore, the economic feasibility of large-scale production of natural coagulants needs further exploration to compete with traditional chemical options.

Future research should focus on optimizing extraction processes, developing industry-wide standards, and exploring hybrid systems that combine the advantages of both natural and chemical coagulants. Addressing these challenges will unlock the full potential of natural coagulants, offering a sustainable and cost-effective solution to meet the growing global demand for clean water while minimizing environmental impact.

## Data Availability

No datasets were generated or analysed during the current study.
